# Unveiling the Neural Environment in Cancer: Exploring the Role of Neural Circuit Players and Potential Therapeutic Strategies

**DOI:** 10.3390/cells12151996

**Published:** 2023-08-03

**Authors:** Tuan Minh Nguyen, Dinh Thi Minh Ngoc, Jung-Hye Choi, Chang-Hoon Lee

**Affiliations:** 1 College of Pharmacy, Dongguk University, Goyang 10326, Republic of Korea; nmtuan28@dgu.ac.kr (T.M.N.); minhngoc@dgu.ac.kr (D.T.M.N.); 2 College of Pharmacy, Kyung Hee University, Seoul 02447, Republic of Korea

**Keywords:** neoneurogenesis, nerve–cancer crosstalk, prognosis, neurotransmitter

## Abstract

The regulation of the immune environment within the tumor microenvironment has provided new opportunities for cancer treatment. However, an important microenvironment surrounding cancer that is often overlooked despite its significance in cancer progression is the neural environment surrounding the tumor. The release of neurotrophic factors from cancer cells is implicated in cancer growth and metastasis by facilitating the infiltration of nerve cells into the tumor microenvironment. This nerve–tumor interplay can elicit cancer cell proliferation, migration, and invasion in response to neurotransmitters. Moreover, it is possible that cancer cells could establish a network resembling that of neurons, allowing them to communicate with one another through neurotransmitters. The expression levels of players in the neural circuits of cancers could serve as potential biomarkers for cancer aggressiveness. Notably, the upregulation of certain players in the neural circuit has been linked to poor prognosis in specific cancer types such as breast cancer, pancreatic cancer, basal cell carcinoma, and stomach cancer. Targeting these players with inhibitors holds great potential for reducing the morbidity and mortality of these carcinomas. However, the efficacy of anti-neurogenic agents in cancer therapy remains underexplored, and further research is necessary to evaluate their effectiveness as a novel approach for cancer treatment. This review summarizes the current knowledge on the role of players in the neural circuits of cancers and the potential of anti-neurogenic agents for cancer therapy.

## 1. Introduction

The tumor tissue of cancer patients is composed of cancer cells and the surrounding microenvironment of neighboring cells. Immunotherapies that regulate the immune milieu within the tumor ecosystem have paved the way for cancer treatment. They present an essential example of controlling the tumor microenvironment in anticancer therapy. However, one often overlooked microenvironment surrounding a cancer is the neural milieu surrounding the tumor.

Tumors can stimulate nerve growth in their vicinity by releasing factors such as lymphangiogenesis and neoangiogenesis, a phenomenon known as neoneurogenesis. This neoneurogenesis may facilitate the development of new tumors, as nerve endings that infiltrate the tumor can release neurotransmitters that promote tumor progression and metastasis [1]. The presence of nerve cell markers in tumor tissue is a predictor of cancer progression, and emerging evidence suggests that the neurotransmitter norepinephrine can enhance cancer dissemination in preclinical models [2]. Although the exact mechanisms underlying neoneurogenesis and its effects on cancer remain incompletely understood, targeting this process represents a promising approach for cancer therapy. This review provides an overview of neoneurogenesis in cancer and its potential role as a therapeutic target.

## 2. Neurogenesis and Neoneurogenesis

### 2.1. Neurogenesis of Nerve Stem Cells (NSCs)

**Figure 1:**
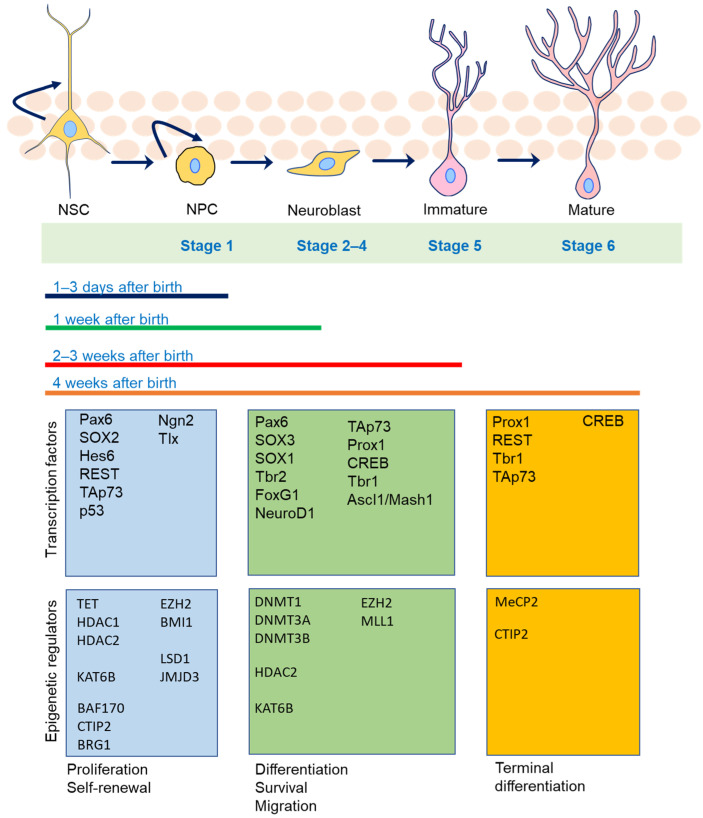
This diagram illustrates the key steps involved in neurogenesis, as well as the role of transcription factors and epigenetic mechanisms in regulating this process. Additionally, it depicts the expression patterns of major epigenetic regulators involved in adult neurogenesis, which play a critical role in determining the fate of neurons during neurogenesis.

Neurogenesis is a complex and highly regulated process that can be divided into six distinct stages [3]. NSCs have two essential properties: self-renewal and multipotency. They can divide and generate more stem cells (self-renewal) while also being able to differentiate into various cell types within the nervous system (multipotency). NSCs can give rise to neurons, astrocytes, and oligodendrocytes [4]. NSCs can differentiate into neuronal progenitor cells (NPCs) at the beginning of hippocampal neurogenesis [5]. Stage 1, known as the proliferation phase, occurs within 1–3 days after birth in mice, during which NPCs can proliferate and differentiate. The differentiation phase (Stages 2–4) follows, beginning approximately 1 week after birth, during which neuronal progenitors cease dividing and become committed to the neuronal lineage. After commitment, the immature neurons enter the migration phase (Stage 5), reaching their destination between 2 and 3 weeks after birth. At this point, post-mitotic neurons extend axonal projections, and dendritic growth commences. The final stage of adult neurogenesis (Stage 6) occurs around 4 weeks after birth, during which newly generated neurons establish synaptic contacts within pre-existing circuits [6,7]. The full integration and incorporation of adult-born neurons into the hippocampal circuits takes approximately 2–4 months [8,9]. All stages are regulated by several main transcription factors and epigenetic regulators [10]. Figure 1 illustrates these stages and epigenetic regulators.

### 2.2. Prognostic Importance of Neoneurogenesis in Cancer

The presence of nerve cells within tumors has been increasingly recognized as a potential factor influencing cancer outcome. This phenomenon is particularly prevalent in cancers of highly innervated organs, including almost all pancreatic cancers, 80% of head and neck cancers, 75% of prostate cancers, and one third of cancers in other organs [11]. The role of nerves in cancer progression is not fully understood, but some studies have suggested that denervation may reduce tumor growth [12,13,14].

The discovery of neoneurogenesis in prostate cancer was among the earliest documented [15]. Moreover, the stimulation of sympathetic nerves in breast cancer has been shown to increase its growth and progression, while the stimulation of parasympathetic nerves has the opposite effect [16,17]. These results suggest that sympathetic and parasympathetic nerve innervation play distinct roles in cancer development. Autonomic nerve development contributes to prostate cancer progression [18] and lung cancer [19], while neurogenesis in colorectal cancer contributes to poor outcome [20].

In addition to the parasympathetic and sympathetic nerves, sensory nerves have also been implicated in tumor progression. For instance, sensory nerves can trigger inflammation and accelerate the development of pancreatic cancer through neurogenic mechanisms [21,22]. During the early stages of pancreatic cancer, there is a shift in the expression of pancreatic neurotrophic factors and an increase in sensory innervation. Furthermore, in one study, nerve growth factor (NGF) knockdown effectively suppressed Panc-1 cells and tumor progression in three pancreatic tumor models, including a subcutaneous model, an orthotopic model, and a patient-derived xenograft model [23]. Later on, cells of pancreatic origin can migrate to the sensory ganglia and spinal cord, indicating that sensory nerves are involved in all stages of pancreatic cancer, from tumorigenesis to progression [24].

Sensory neurons have also been shown to play a direct role in tumor formation in basal cell carcinoma [25]. In the stomach, vagotomy or the pharmacological denervation of the portion containing both parasympathetic and sensory axons reduced tumor progression and improved survival when performed in the later stages of the disease [26]. This denervation specifically attenuated gastric tumors and enhanced the effects of chemotherapy [27]. Additionally, enteric nerves are implicated in gastric cancer initiation and progression [28,29].

## 3. The Relationship between Neurogenesis and Cancer

**Figure 2:**
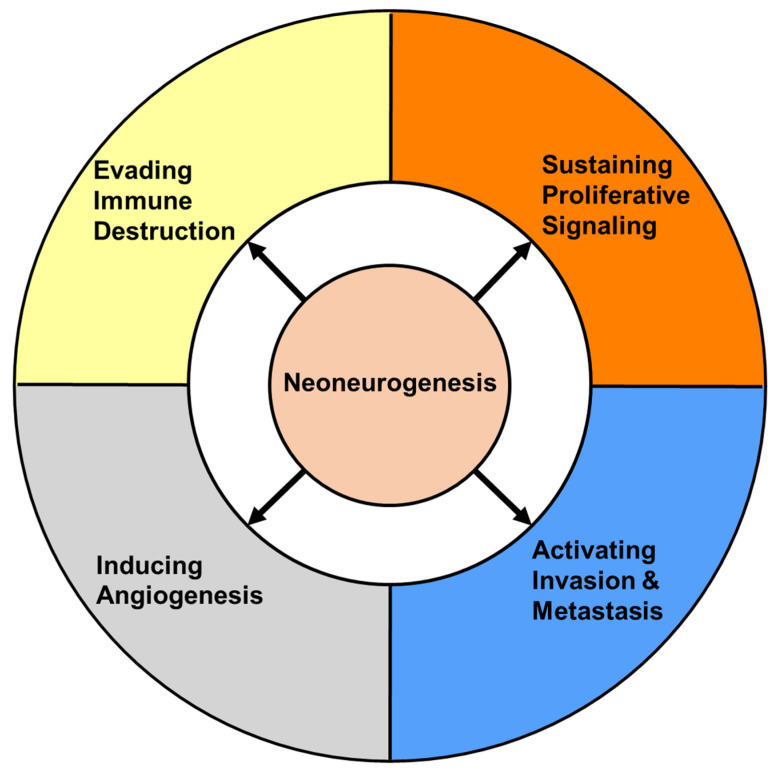
Effects of neoneurogenesis on cancer progression. This illustration encompasses four experimentally determined effects of neoneurogenesis on the hallmarks of cancer: neoneurogenesis has been found to sustain proliferative signaling in prostate cancer [18], skin cancer [30], breast cancer [31], and brain cancer [32]; activate invasion and metastasis in prostate cancer [18], brain cancer [33], and lung cancer [34]; induce angiogenesis in breast cancer [35], gastric cancer [36], and prostate cancer [37]; and evade immune destruction in breast cancer [38] and pancreatic cancer [39]. The separate hallmarks of cancer are depicted in differently colored fields.

In recent years, the interaction between cancer cells and neurons within the tumor microenvironment has been recognized. Specifically, the effects of “cancer on neurons”, “neurons on cancer”, and “neurons on tumor microenvironment” have been a focus of investigation. These relationships suggest that neoneurogenesis may play a critical role in regulating certain hallmarks of cancer (see Figure 2).

### 3.1. The Effects of Cancer on Nerves

Cancer cells can engage in neoneurogenesis and recruit new axons into tumor tissue, which is a process similar to cancer angiogenesis. This process is called neoneurogenesis, also known as innervation, and is a complex biological phenomenon that is not yet fully understood [40]. Neoneurogenesis arising from different nerves may play different or even opposite roles in different types of tumors.

Compellingly, some cancer stem cells (CSCs) obtained from individuals with gastric and colorectal carcinoma were found to be capable of generating neurons that contribute to tumor neurogenesis and tumor growth [41]. By isolating a single cancer stem cell and creating a clone, researchers demonstrated that this clone could generate various types of neurons, such as sympathetic and parasympathetic neurons, which become part of the nervous system within cancerous tissues [41]. When the capacity of these CSCs to produce neural cells was suppressed, the growth of xenograft tumors in a mouse model was inhibited [41]. These findings support that new neurons could be generated by cancer.

The peripheral nervous system (PNS), which includes sympathetic and parasympathetic nerves, helps to maintain the body’s homeostasis. The neurotransmitter of the sympathetic nerves is norepinephrine, while that of the parasympathetic nerves is acetylcholine, both of which play crucial roles in cellular communication. These interconnected systems regulate the body’s internal pressures [42]. Sympathetic and parasympathetic nerves often have opposing effects on a given tissue, increasing the activity of one system while decreasing the activity of the other. Neoneurogenesis in tumors has been linked to the PNS, which includes both sympathetic and parasympathetic nerves, responding to changes in the microenvironment [43].

Neural progenitors expressing (DCX+) from the central nervous system infiltrate prostate tumors and metastases, initiating neurogenesis [44]. In mouse models of prostate cancer, oscillations of DCX+ neural progenitors in the subventricular zone (a neurogenic area) disrupt the blood–brain barrier, allowing DCX+ cells to enter circulation. These cells then infiltrate the tumor, generating new adrenergic neurons. Genetically removing DCX+ cells inhibits early tumor development in mouse models, while transplanting DCX+ neural progenitors promotes tumor growth and metastasis [44]. In humans, the density of DCX+ neural progenitors strongly correlates with the aggressiveness and recurrence of prostate adenocarcinoma. [44]. These findings demonstrate a unique interaction between the central nervous system and prostate tumors, providing potential neural targets for cancer treatment.

Recent findings suggest that newly formed adrenergic nerve fibers in head and neck cancers are derived from sensory neurons and are not infiltrations of existing adrenergic nerves [45]. Thus, signals that stimulate tumor growth are regulated by newly formed adrenergic nerve fibers instead of pre-existing ones [45]. In one study, MiR-34a released in extracellular vesicles impeded neuron filament generation and was downregulated by p53 loss [45]. MiR-34a prevents somatic cell programming, neuronal differentiation, and development [46,47,48]. The reprogramming of sensory nerves caused by a lack of miR-34a in p53 null head and neck cancer-derived exosomes has been found to lead to tumor progression [45]. Blocking adrenergic receptors through sensory denervation or pharmacological means suppresses tumor growth, while the chemical sympathectomy of preexisting adrenergic nerves does not [45]. The research indicates that cancer cells drive neuron reprogramming to promote tumor progression. However, the potential role of neuron reprogramming induced by cancer cells in other types of tumors remains to be determined.

### 3.2. The Effects of Nerves on Cancer

The crosstalk between cancer cells and nerves in the tumor microenvironment has been identified as an important factor in tumor progression. In this section, we focus on the impacts of the “nerves on cancer” relationship, which can be broadly categorized into two primary modes: paracrine signaling and chemical synapses.

#### 3.2.1. Paracrine Signaling

Paracrine signaling is the methods by which cells communicate with nearby cells through signaling molecules that bind to and activate surrounding cells [49]. Neurons and Schwann cells can regulate cancer cell behavior and impact tumor progression through paracrine signaling using neuroactive substances. The neuroactive substances secreted by nerves can be broadly categorized into three groups: (i) neurotrophic factors, including nerve growth factor (NGF), brain-derived neurotrophic factor (BDNF), and other factors; (ii) axon guidance molecules, such as CCL2, CX3CL1, and others; and (iii) neurotransmitters like acetylcholine (Ach), glutamate, glycine, and others [50,51]. These molecules interact with receptors expressed by cancer cells, such as TrkA, TrkB, and NGFR.

In prostate cancer, for example, adrenergic fibers newly extended from nerves regulate β2- and β3-adrenergic receptors in cancer, while cholinergic fibers act through cholinergic receptors [18]. The genetic deletion of sympathetic β2- and β3-adrenergic receptors in stromal cells has been found to prevent early tumor progression. In contrast, parasympathetic stimulation contributes to later tumor progression, invasion, and metastasis through the pharmacological or genetic disruption of the muscarinic 1 receptor [18,52]. In skin and breast cancers, adrenergic antagonists have been shown to have a suppressive effect on cancer development [30,31,53].

Nerves that stimulate the release of ACh through cholinergic stimulation can enhance the expression of NGF in the gastric epithelium, leading to the advancement of cancer [29]. β-blockers can also increase the rate of survival in prostate cancer patients undergoing high-risk or metastatic treatment [54].

#### 3.2.2. Chemical Synapse

Chemical synapses are identified as biological junctions, which are referred to as the synaptic cleft, between the plasma membranes of the interconnected cells [55]. The transfer of information between the presynaptic and postsynaptic cells of neurons or non-neuronal cells (muscles or glands) is facilitated by a molecule called the neurotransmitter [55]. Chemical synapses allow neurons to form circuits within the central nervous system [56]. The chemical synapse also forms crosstalk between tumors and nerves in which adjacent neurons communicate through neurotransmitters such as glutamate [57]. Evidence of functional synapses between presynaptic neurons and postsynaptic tumor cells has been observed in glioma [58,59] and breast-to-brain metastasis [33]. These neurogliomal synapses may be functional, as excitatory postsynaptic potentials have been recorded in glioma cells [58,59]. Studies have shown that the AMPA receptor promotes the depolarization of glioma cells, while the NMDA receptor promotes the growth of cancer cells in the brain [32,60]. High levels of the NMDA receptor subunit GLuN2B were observed in breast-to-brain metastasis (B2BM), and after NMDAR activation, currents and calcium transients were recorded [33,61,62]. The knockdown of GLuN2B resulted in smaller brain tumors and longer survival times in mice, suggesting that NMDAR synapses promote the growth of cancer cells in the brain [33]. However, it is still unknown whether other solid tumors form synapses with nerves.

In summary, the impacts of nerves on tumors are complex and multi-faceted and involve both the paracrine mode and the chemical synapse. Understanding the mechanisms of these interactions is important for the development of new therapeutic strategies for cancer.

### 3.3. The Effects of Nerves on the Tumor Microenvironment

Beyond the well-established effects of neurons on cancer development via paracrine signaling and chemical synapses, emerging evidence suggests that neurons can also significantly impact cancer progression by manipulating the tumor microenvironment. Studies have shown that neurons can promote angiogenesis [35,36,37,63,64] and modulate tumor immune environments to create a more favorable environment for breast, lung, and pancreatic cancer growth [34,38,39] and lung cancer metastasis [34].

#### 3.3.1. The Effects of Nerves on Angiogenesis

Angiogenesis, the process by which new blood vessels grow from existing vasculature, is critical for tumor growth and metastasis [65,66]. Tumors require their own sources of oxygen and nutrients to sustain cell proliferation and tumor growth. The degree of angiogenesis reflects the severity of a tumor, and its presence has been linked to the outcomes of tumors [67,68]. The neurotransmitters and neurotrophic factors released by nerves play a role in angiogenesis by binding to receptors and triggering the migration of endothelial cells. Examples of these factors include catecholamines, acetylcholine, dopamine, nerve growth factor, and brain-derived neurotrophic factor [35,36,63]. Studies have shown that adrenergic nerves regulate angiogenesis in the prostate cancer microenvironment by altering the metabolism of blood vessel endothelial cells, leading to tumor growth through angiogenesis [37]. The regulation of angiogenesis and neoneurogenesis share similarities, including being controlled by the same transmitters and neurotrophic factors and using similar receptors [64]. These findings highlight the close relationship between the regulation of angiogenesis and neurogenesis.

#### 3.3.2. The Effects of Nerves on the Immune System 

The nervous system interacts with the immune system within the tumor microenvironment (TME) to promote tumor progression through inflammation [69]. Neuroendocrine and neuronal pathways play a role in controlling immune responses [70]. For example, adrenergic innervation in the spleen stimulates the production of ACh in T cells that express the β2-adrenergic receptor (β2-AR) [71]. Recent research has revealed that ACh produced by T cells has a significant impact on the regulation of immunity, including cancer immunity. This ACh can inhibit the production of tumor necrosis factor (TNF) by cytokine-producing macrophages through the α7 nicotinic acetylcholine receptor [72]. ACh also binds back onto the nicotinic and muscarinic receptors on lung cancer cells, which leads to an acceleration in cell proliferation, migration, and invasion [34]. Choline acetyltransferase catalyzes the synthesis of ACh from choline, which is strongly induced in both CD4+ and CD8+ T cells through IL-21 to regulate T-cell migration and immune functions [73]. This research highlights how the autonomic nervous system can directly regulate the immune system.

The infiltration and activation of tumor lymphocytes are critical processes for inhibiting tumor growth and progression [74]. However, tumor cells can evade immunosurveillance by activating immune checkpoint pathways that suppress antitumor immune responses [75]. A retrospective analysis of breast cancer patients revealed that sympathetic and parasympathetic nerve density correlates with the expression of immune checkpoint molecules (PD-1, PD-L1, and FOXP3) and clinical outcomes [38].

Tumor-associated macrophages (TAMs) play critical roles in regulating pancreatic tumor development and progression as essential components of the cancer microenvironment [76]. TAMs can also modify tumor cell resistance to chemotherapy through their impact on the TME [77]. The recruitment of TAMs is also regulated by both cholinergic and adrenergic signaling, which are related to nerves. In pancreatic cancer, adrenergic signaling promotes tumor growth and reduces survival through TAM recruitment, while cholinergic signaling has the opposite effects [39]. One study showed that vagotomy promoted pancreatic cancer growth and reduced survival time by mediating TNFα secretion by TAMs [78]. Similar results have been observed in breast cancer [78]. Stress-induced neuroendocrine activation also causes breast cancer metastasis, and this can be reversed by a beta antagonist [79]. Endoneural macrophages participate in tumor metastasis, and blocking certain pathways can inhibit brain metastasis [80]. The elimination or inhibition of microglia function results in a good antitumor metastasis effect. The blocking of any of the CCL2, STAT3, CSF-1R, and PI3K pathways of macrophages could inhibit brain metastasis [81,82,83]. In summary, nerves can impact tumor progression by regulating the behavior of immune cells.

## 4. The Neural Circuit of Cancer

**Figure 3:**
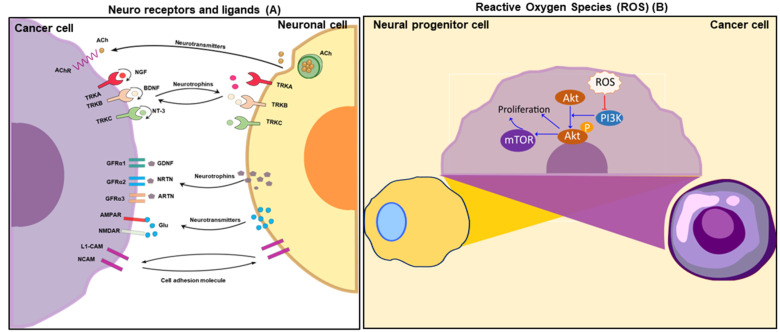
Illustration of the characteristics shared between neurons and cancer cells. (**A**) This diagram highlights the importance of reciprocal signaling between the two cell types for neurogenesis, axonogenesis, and perineural invasion. (**B**) The ROS/PI3K/AKT/mTOR pathway is involved in proliferation in neurons and cancer cells.

Mounting evidence suggests that neural factors may play a role in promoting cancer development. To support this idea, studies have found that many neuron receptors, their ligands, and proteins highly expressed in neurons are also significantly expressed in cancer cells. Moreover, these proteins have been found to be involved in similar functions in both neurons and cancer, suggesting that cancer cells may acquire the properties of neuronal cells, potentially explaining the similarities in behavior between these neurons and cancer. This phenomenon is discussed below and illustrated in Figure 3.

This phenomenon has led to the hypothesis that tumor cells may undergo a transformation into neuron-like cells and establish a neural circuit, allowing cancer cells to communicate with neuron cells and other cancer cells and mimic the survival and metastatic properties of neurons. This idea is supported by the upregulation of neuronal receptors such as AMAPR, NMDAR, GFRα1, GFRα2, GFRα3, AChR, L1-CAM, and NCAM and their ligands, including glutamine, GDNF, NRTN, ARTN, and Ach, in cancers (Figure 3).

### 4.1. Receptors in the Neural Circuit of Cancers

In recent studies, it has been discovered that certain tumor types show a high level of expression of neuron receptors such as Trks, NGFR, GFRα, L1CAM, NCAM, AchRs, AMPARs, NMDAR, and dopamine receptors. This discovery provides an explanation for the connection between neurons and cancer (Figure 3A) and suggests there is a cancer network, allowing cancer cells to be capable of communicating with one another in a way that is similar to neurons communicating in a neuronal circuit.

#### 4.1.1. Neurotrophic Receptor Tyrosine Kinase (Trks)

The Trk receptor family comprises three receptors, TrkA, TrkB, and TrkC, which are situated on the cell membrane [84]. These receptors are high-affinity neurotrophin receptors [85] and are activated by neurotrophins [86], which are a type of growth factor essential for proper nervous system function [87]. Trks bind to nerve growth factors (NGFs), such as brain-derived neurotrophic factor (BDNF) and NGF, which are secreted by nerves and cancer cells, and promote cancer cell survival, migration, and proliferation [88]. Trk genes have been identified in several solid tumors such as lung cancer [89]. In gastric cancer, expanded enteric nerves and increased NGF expression are associated with the NGF/Trk signaling regulation of microtubule-associated doublecortin-like kinase 1 (DCLK1), also known as the tuft cell marker in normal tissue or the tumor stem cell marker in cancers [29,88]. Blocking NGF signaling via NGF knockdown or the NGF-neutralization of antibodies reduces the migration of pancreatic cancer cells toward the dorsal root ganglia, while breast cancer cells drive axonogenesis in PC12 cells through the correlation between nerve fibers and NGF expression, a process partly reversed by NGF blocking [90,91].

#### 4.1.2. Nerve Growth Factor Receptor (NGFR)

The nerve growth factor receptor (NGFR), also known as p75, is a low-affinity receptor for all known mammalian neurotrophins (i.e., proNGF, NGF, BDNF, NT-3 e NT-4/5) [92], and it is involved in pathways that determine both the survival and death of neurons [93]. NGFR is expressed in various cancer types, including luminal breast cancer in rare basal-like cells that are resistant to antiestrogens [94]. NGFR inhibits p53 activity in tumor cells via a negative feedback loop present in multiple tumor types, which is critical for maintaining melanoma stem cells in vitro and melanoma growth in vivo [95]. TNBC cells rely on the significant upregulation of NGFR expression to facilitate their growth as tumor spheres in a non-adhesive environment. This elevated expression was shown to play a crucial role in enabling the cells to evade anoikis (cell death triggered by detachment), support the growth of the primary tumor, and enhance metastasis in mice [96]. Thus, NGF signaling from nerves to cancer stem cells via NGFR expression may promote cancer stem cell renewal and proliferation.

#### 4.1.3. Glial Cell Line-Derived Family Receptor Alpha (GFRα) Family

The GFRα family, consisting of GFRα1, GFRα2, GFRα3, and GFRα4, is anchored to the plasma membrane via Glycosylphosphatidylinositols (GPIs). GPIs serve as membrane anchors for numerous eukaryotic cell surface proteins, including members of the GFRα family [97]. Although GFRα1, GFRα2, GFRα3, and GFRα4 are structurally similar, they determine specificity for four ligands called glial cell line-derived neurotrophic factor (GDNF), neurturin (NRTN), artemin (ARTN), and persephin (PSPN), respectively [98]. GFRα receptors, which are associated with cell growth, differentiation, cell migration and tissue maturation, have been extensively linked to various cancers [97]. GFRα1 expression is significantly upregulated in human breast cancers [99]. GFRα1 is involved in chemoresistance in osteosarcoma [100,101] and prostate cancer [102]. GFRα2 promotes neuroblastoma cell proliferation by activating the PI3K/AKT pathway [103]. The GFRα3 promoter region has been shown to be markedly hypermethylated in almost all gastric tumors [104]. GDNF promotes cancer progression by binding to GDNF family receptors α1-3 and RET proto-oncogene (RET), which initiate the downstream activation of several signaling pathways, including RAS/ERK, MAPK, JNK, and PI3-K/Akt [105,106].

#### 4.1.4. L1 Cell Adhesion Molecule (L1CAM)

The L1 cell adhesion molecule (L1CAM) is a cell surface receptor that plays a key role in cell adhesion and migration during neural development. L1CAM is upregulated in various types of tumors and enhances cancer cell invasiveness, leading to metastasis and resistance to chemo- and radiotherapy [107,108]. Additionally, L1CAM expression, together with CD133 expression, defines the cancer stem cell population in glioma and ovarian cancer. In vivo ovarian cancer models have shown that L1CAM expression promotes cancer stemness by enhancing spherogenicity, tumor take rate, self-renewal capacity, and tumor growth [108].

#### 4.1.5. Neural Cell Adhesion Molecule (NCAM)

Neural cell adhesion molecules (NCAMs) are a type of glycoprotein found on the surface of cells in both the central and peripheral nervous systems [109]. They have a wide range of isoforms due to them having at least 27 alternatively spliced NCAM mRNAs. The three main isoforms of NCAMs vary only in their cytoplasmic domain; they include NCAM-120kDa (GPI anchored), NCAM-140kDa (short cytoplasmic domain), and NCAM-180kDa (long cytoplasmic domain) [110]. NCAMs are involved in a diverse range of contact-mediated interactions among neurons, astrocytes, oligodendrocytes, and myotubes. NCAMs have been known to be involved in lung cancer cases with poor prognoses [111]. NCAMs have also been found to promote cell migration and invasion in ovarian cancer [112].

#### 4.1.6. Acetylcholine Receptors (AChRs)

Acetylcholine (ACh) is a neurotransmitter that plays various roles in the central nervous system, peripheral nervous system, and autonomic nervous system. ACh acts on two types of receptors—muscarinic receptors (mAChRs) and nicotinic receptors (nAChRs) [113]. The family of mAChR comprises five subtypes, which are labeled individually as M1 to M5 and are encoded by the genes CHRM1 to CHRM5. Among these subtypes, M1, M3, and M5 have been observed to interact with G proteins of the Gq/11 family, whereas M2 and M4 primarily signal through the Gi/o family of G proteins [114]. Meanwhile, nAChRs are composed of five subunits which maintain a particular arrangement around a water-filled pore. These subunits can be classified into two categories: alpha (α2–α7, α9, and α10) and beta (β2–β4), with the classification being based on the presence of adjacent cysteine groups that are exclusive to the α subunits’ extracellular domain in neurons [115].

Recent studies have suggested that the overexpression of AChRs in cancer cells contributes to cancer cell proliferation, apoptosis, angiogenesis, and even epithelial-mesenchymal transition (EMT) [116]. In breast cancer, alpha7-nAChR and alpha9-nAChR serve as oncogenes [116]. Moreover, non-neuronal cells, such as immune cells [117,118] and cancer cells [119,120], are capable of synthesizing ACh, leading to autocrine and paracrine effects in various tumor microenvironments [121].

#### 4.1.7. AMPA, NMDA and Metabotropic Glutamate Receptors

AMPA receptors (AMPARs) are receptors for glutamate and have a type of ion channel composed of four subunits encoded by different genes, designated as GRIA1, GRIA2, GRIA3, and GRIA4, which combine to form tetrameric heteromeric complexes [122]. AMPARs are responsible for most excitatory communication in the central nervous system, along with other ionotropic glutamate receptors like NMDA and kainate receptors [123]. The specific properties of AMPARs, such as their kinetics and conductance, are determined during their formation and can be influenced by various factors such as post-transcriptional RNA editing, splice variation, post-translational modification, and the subunit makeup of the receptors [123]. Glioma cells express AMPA receptors in large quantities, and these receptors play a significant role in promoting the malignant properties of the cancer by responding to glutamate signals that facilitate cell proliferation [124,125]. AMPA receptors have also been found in pancreatic cancer, non-CNS cancers [126].

A NMDA receptor (NMDAR) is a receptor protein for glutamate and has an ion channel that is activated upon binding to glycine and glutamate [127]. The receptor is typically comprised of heterotetramers made up of two NR1 subunits and two NR2 subunits, according to reference [128]. Peripheral cancers mainly exhibit NMDA receptor expression. Prostate cancer samples have shown moderate-to-high levels of the NR1 subunit of NMDAR, while normal prostate tissue and benign prostate hyperplasia have very low or no expression. A similar pattern of expression has been observed in normal vs. cancer colon specimens [129]. The presence of the NR1 subunit has also been observed in most small-cell lung cancer samples and breast cancer samples [130]. Additionally, the NR2B subunit has been detected in breast cancer samples [131]. Inhibiting NMDA receptors through the use of the channel-blocking antagonist MK-801 may reduce pancreatic cell viability [132]. Moreover, the NMDA antagonist dizocilpine can inhibit the extracellular signal-regulated kinase 1/2 (ERK1/2) pathway, an intracellular signaling cascade that is stimulated by growth factors and governs the proliferation of cancer cells [133]. NMDA is involved in regulating mTOR signaling, which suggests that NDMA might regulate cancer cell growth, division, and invasiveness [134]. 

Metabotropic glutamate receptors (mGluRs) belong to the group of glutamate receptors that engage in an indirect metabotropic mechanism. These receptors are classified as G-protein-coupled receptors (GPCRs) and are part of the Group C family of GPCRs [135]. mGluRs can affect the transformation of peripheral cells and the growth of tumors. This can occur through various mechanisms, such as the ectopic expression of normal mGluRs, the generation of increased proliferative signals from the overexpression of the receptors, mutations in the receptors, or the expression of polymorphic variants [136].

These receptors’ expression in cancer cells suggests that glutamate signaling might play a vital role in cancer cell survival and proliferation.

#### 4.1.8. Dopamine Receptors

Dopamine receptors are a type of G-protein-coupled receptor that play a significant role in the central nervous systems (CNSs) of vertebrates [137]. Dopamine receptors can activate various effectors not only through G protein coupling but also by interacting with different proteins such as dopamine-receptor-interacting proteins [138] There are two subtypes of D1-like receptors (D1 and D5) that are linked to the G protein Gs and activate adenylyl cyclase (AC), while the other subtypes belong to the D2-like subfamily (D2, D3, and D4) [139]. D2-like receptors exhibit a significantly higher affinity for dopamine compared to the D1-like receptor family, ranging from 10 to 100 times greater [140]. 

The activation of the D2-like receptor family typically results in the inhibition of adenylyl cyclase (AC) activity, as well as the inhibition of protein kinase A (PKA) and DARPP-32 [141]. Additionally, D2Rs modulate G-protein-coupled inward rectifier potassium (GIRK) channels, which are responsible for mediating neuronal electrical responses [142]. D2Rs also have the ability to activate pathways associated with cell proliferation, such as the mitogen-activated protein kinase (MAPK) signaling pathway. The activation of ERK1/2, a component of MAPK, has been observed in various cell lines, including HEK-293 cells and COS-7 cells [141]. The stimulation of D2-like receptors also triggers signaling through the Akt pathway, also known as protein kinase B (PKB) [143,144].

DRD2 (a member of D2-like family), which is encoded by the DRD2 gene, is a receptor for the neurotransmitter dopamine and a target for antipsychotic drugs as well as Parkinson’s disease treatment. It is activated by dopamine and synthetic agonists such as bromocriptine, leading to the activation of Gi and inhibition of adenylyl cyclase [145]. Additionally, DRD2 is a key component of the dopamine signaling pathway that is involved in normal growth and development, as well as cancer development [146]. Studies have shown that DRD2 is overexpressed in various types of cancer, including breast, ovarian, cervical, esophageal, and lung cancers [146]. Furthermore, research has demonstrated that DRD2 knockdown in HCT116 cells activates the integrated stress response and reduces cell proliferation [147]. DRD2 activation has been shown to promote self-renewal in breast cancer by activating STAT3 and IL-6 [148].

### 4.2. Ligands in the Neural Circuits of Cancers

The identification of neuroactive substances such as GDNF, NRT, and glutamate being released by cancer cells provides evidence for the notion that these cancerous cells engage in communication through a circuitry that involves the release and reception of neuronal signals, much like in a neuronal network (Figure 3A).

#### 4.2.1. Brain-Derived Neurotrophic Factor (BDNF), Neurotrophin-3 (NT-3), and Nerve Growth Factor (NGF)

NGF plays a crucial role in the growth and preservation of neurons in the peripheral nervous system (PNS), as well as in the functional maintenance of cholinergic neurons in the central nervous system (CNS) [149]. The nociceptive signals are transmitted through NGF by directly activating TrkA [150]. The NGF/TrkA pathway has also been implicated in the development and progression of breast cancer [151,152] and prostate cancer [153,154]. NGF has been identified as a factor released by mouse sarcoma tissue, promoting the survival of neurons and the growth of nerve extensions (neurite outgrowth) in chicken ganglia [155].

BDNF is produced in response to noradrenergic signaling and activates axonogenesis via TrkB receptors [156,157]. In addition to its role in axonogenesis, BDNF has been linked to angiogenesis promotion and increased tumor cell proliferation, which may contribute to the progression of pancreatic ductal adenocarcinoma (PDAC) [158]. Targeting BDNF signaling pathways may hold promise as a strategy for inhibiting PDAC progression and neural invasion. Moreover, BDNF is released from breast cancer cells [159] and promotes breast cancer progression [159,160].

NT-3 is most highly expressed in immature regions of the CNS [161], which is a cystine knot growth factor by activating TrkC [162] that promotes the survival, proliferation, and differentiation of developing neurons, and is considered a potential therapy for neurodegenerative disorders [163]. In murine xenograft models, the inhibition of NT-3 has been demonstrated to reduce PDAC growth [88]. Interestingly, NT-3 is secreted from metastatic breast cancer in the brain as MDA-MB-361 and in triple-negative breast cancer as MDA-MB-231 [164]. NT-3 modulates the growth of breast cancer brain metastasis and breast cancer cells by interacting with the microenvironment [164]. In neuroblastomas, the overexpression of NT-3 and TrkC is linked to a poor prognosis [165]. 

#### 4.2.2. Glial Cell-Derived Neurotrophic Factor (GDNF), Neurturin (NRTN), and Artemin (ARTN)

Glial cell line-derived neurotrophic factor (GDNF) is a glycosylated, disulfide-bonded homodimer that is a distantly related member of the transforming growth factor-beta superfamily [166] GDNF is a small protein that potently promotes the survival of many types of neurons [167]. GDNF is highly expressed in human PDAC and is strongly correlated with neural invasion and increased pain levels [105,168]. These pathways promote cell proliferation, survival, and migration, which contribute to the spread of cancer cells. GDNF also promotes nerve adhesion and invasion by increasing integrin expression, activating matrix metalloproteinase (MMP)-9 and increasing nuclear factor κ B activity [169,170]. Targeting GDNF or its receptors also may be a promising approach for inhibiting PDAC progression and neural invasion.

Neurturin (NRTN) is a substance that helps neurons survive by activating the Ret tyrosine kinase when a GPI-linked coreceptor such as GFRα1 or GFRα2, is present [171]. Moreover, NRTN mRNA expression is upregulated in some cancers, such as kidney renal papillary cell carcinoma (KIPR) and ovarian cancer (OV) from TCGA [172].

Artemin (ARTN), a new member of the GDNF family, has been discovered to be the ligand for the GFRalpha3-RET receptor. Artemin promotes the survival of both sensory and sympathetic neurons when cultured, and based on its pattern of expression, it is believed to have an effect on these types of neurons in their natural setting as well [173]. The stimulation of RET signaling promotes innervation [174] and is associated with tumor invasiveness and nerve alterations in PDAC due to high expression levels of its ligands, such as artemin [175,176,177]. Artemin is primarily detected in hypertrophic nerves using western blotting and in PDAC tissues using immunohistochemistry [178].

#### 4.2.3. Glutamate

Glutamate is an excitatory neurotransmitter found in different types of receptors such as AMPA and NMDA receptors within the central nervous system, and maintaining optimal levels of it in the extracellular space is crucial [179,180]. Glutamate plays a significant role in the timing of various events, including the survival, proliferation, migration, synapse formation, and integration of newly formed neurons into established synaptic networks [181]. Glioblastoma cells can release glutamate excessively [124]. Compellingly, glutamate has been found to be released by non-CNS cancers, such as MDA-MB-231 (human breast cancer), B16F1 (mouse melanoma), and MATLyLu (rat prostate cancer) [182,183]. Glutamate increases pancreatic cancer cell invasion and migration via AMPA receptor activation and Kras-MAPK signaling through AMPA [126].

#### 4.2.4. Acetylcholine

Acetylcholine operates through two subgroups of receptors known as muscarinic receptors (mAChRs) and nicotinic receptors (nAChRs) [116]. Additionally, human colon cancer cells were found to release ACh, which in turn stimulated cell proliferation through autocrine stimulation [119], and ACh was also observed to be released by lung carcinoma (H82 cells) [120]. It is speculated that cancer cells could use ACh and AChR in neuronal circuits to communicate with one another.

#### 4.2.5. Dopamine

Dopamine, also known as 3-hydroxytyramine, is part of the catecholamine group and is primarily recognized as a neurotransmitter in the central nervous system [184]. Furthermore, dopamine secretion was found to increase in human hepatocellular carcinomas (HCCs) such as HepG2 [185]. According to research, some DRD2 antagonists like penfluridol, pimozide, and fluspirilene have shown significant cancer suppression [186,187,188]. This indicates that dopamine could be involved in cancer progression via binding dopamine receptors being highly expressed in cancers.

### 4.3. Reactive Oxygen Species (ROS) in the Neural Circuits of Cancers

The role of reactive oxygen species (ROS) in cellular processes has been extensively studied due to their harmful effects. Although stem cells were initially believed to maintain low ROS levels to prevent damage, recent studies have shown that ROS can also act as second messengers, activating normal cellular processes during neurogenesis. Moreover, ROS plays a crucial role in cell proliferation and has been associated with poor cancer prognoses [189,190]. These findings suggest that ROS is involved in regulating both neurogenesis and cancer development Figure 3B.

#### 4.3.1. The Role of ROS in Neurogenesis

The stimulation of ROS, such as H_2_O_2_, has been found to enhance neural progenitor cell (NPC) proliferation and differentiation into both neuronal and oligodendrocyte fates [191]. Self-renewing multipotent neural progenitors that share phenotypic characteristics with neural stem cells (NSCs) maintain high ROS levels and exhibit heightened responsiveness to ROS stimulation. The PI3K/Akt/mTOR signaling pathway mediates the promotion of self-renewal and neurogenesis by ROS [192]. However, an overload of ROS during aging impairs adult neurogenesis [193].

#### 4.3.2. The Role of ROS in Cancer

ROS-sensitive signaling pathways are upregulated in several types of cancer, where they play roles in cell proliferation, differentiation, protein synthesis, glucose metabolism, cell survival, and inflammation [194]. H2O2 can regulate the activity of protein targets, such as protein tyrosine phosphatases, protein tyrosine kinases, receptor tyrosine kinases, and transcription factors, through reversible oxidation [194]. ROS also regulates the MAP kinase/Erk cascade [194], PI3K/Akt-regulated signaling cascades [195,196], and the IκB kinase (IKK)/nuclear factor κ-B (NF-κB)-activating pathways in cancers [197]. 

The PI3K/Akt signaling pathway Is activated by ROS generation during estrogen metabolism or other potential mammary carcinogens in breast cancer [195,196]. Akt mediates cell survival through phosphorylation and the inactivation of its substrates, such as pro-apoptotic proteins Bad and Bax and transcription factors [198,199,200,201]. EGF-generated hydrogen peroxide activates Akt and p70 S6K1, a substrate of Akt that regulates protein synthesis, in human ovarian cancer cells [202]. Additionally, reducing ROS levels has been found to decrease phosphorylated (active) Akt levels and induce apoptosis in the human pancreatic tumor cell line Panc-1 [203].

## 5. Modulators of the Neural Circuit as Anticancer Agents

**Figure 4:**
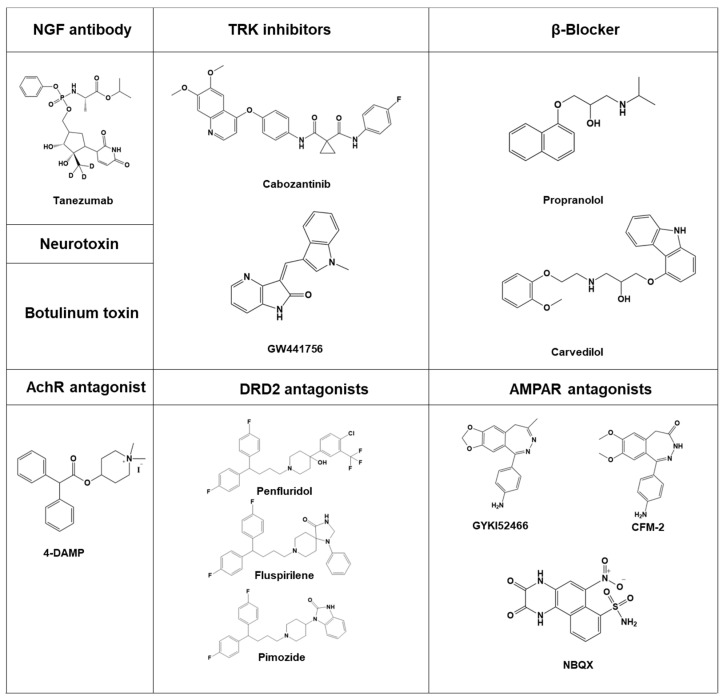
Potential agents that target neurogenesis. These agents include tanezumab, a monoclonal antibody against nerve growth factor; cabozantinib, a medication used to treat medullary thyroid cancer, renal cell carcinoma, and hepatocellular carcinoma; GW441756, a potent, selective inhibitor of the NGF receptor tyrosine kinase A; propranolol, a medication of the β-blocker class used to treat high blood pressure; carvedilol, a β-blocker medication used to treat high blood pressure, congestive heart failure (CHF), and left ventricular dysfunction; A-DMAP, a selective muscarinic acetylcholine receptor (mAChR) M3 antagonist; penfluridol, fluspirilene and pimozide, the first diphenylbutylpiperidine antipsychotics; GYKI 52466, a non-competitive AMPA receptor antagonist; CFM-2, a selective and non-competitive AMPA receptor antagonist which inhibits the ERK1/2 pathway and acts as an antiproliferative agent; and NBQX, an antagonist of the AMPA receptor which blocks AMPA receptors in micromolar concentrations and also blocks kainate receptors.

Inhibiting neurogenesis is a potential strategy for cancer treatment, but currently, there are only a limited number of agents available (Figure 4) that specifically target neurogenesis. Neutralizing NGF antibodies such as tanezumab and fulranumab have been studied and may have the potential to inhibit cancer cell migration with minimal side effects on neural function. A clinical trial (NCT02609828) is currently underway to evaluate the efficacy of these agents [91,204,205]. 

Inhibitors of tyrosine kinase, such as GW441756 and cabozantinib, have been shown to reduce cancer cell migration with minimal impact on cognitive function. One clinical trial (NCT02609828) has been completed, while another (NCT02219711) is still ongoing [91]. Furthermore, ongoing clinical trials (NCT02944201 and NCT03152786) suggest that beta-blockers such as propranolol and carvedilol may increase prostate survival rate and impede cancer cell growth [206,207]. Although Botulinum toxin has been shown to increase apoptosis in cancer cells, a clinical trial with the identifier NCT01520441 was cancelled [208].

In addition, DRD2 agonists have been found to negatively regulate reactive oxygen species (ROS) [209,210]. Research conducted in vivo and in vitro has demonstrated that the protective effects of D2R agonists against oxidative stress are abolished by D2R antagonists [211]. Additionally, a derivative of trifluoperazine, A4, provoked increased ROS, DNA damage, autophagic cell death, apoptosis, and the activation of AMP-activated protein kinase K [212]. 

Interestingly, recent studies have revealed that antipsychotics targeting the D2 receptor have the potential to suppress various types of cancer and increase the levels of reactive oxygen species (ROS) [146]. For instance, penfluridol, a first generation diphenylbutylpiperidine antipsychotic and also a DRD2 antagonist, has been shown to inhibit acute myeloid leukemia through ROS modulation [213]. Similarly, ONC201 treatment resulted in a dose-dependent increase in ROS levels and depolarization of mitochondrial membranes in ovarian cancer cells. This was accompanied by the upregulation of several markers of oxidative stress, including ATF4, CHOP, PERK, and Ero1-1α, all of which are associated with apoptosis [214]. Furthermore, ONC201 has been found to suppress endometrial cancer growth and generate ROS [215]. Pimozide, a DRD2 antagonist which is also a type of diphenylbutylpiperidine antipsychotic, has been shown to inhibit prostate cancer cells via ROS similarly to other diphenylbutylpiperidine antipsychotics [186]. Additionally, pimozide was found to suppress cancer progression through other mechanisms [216,217]. Fluspirilene, another DRD2 antagonist, was also found to suppress hepatocellular carcinoma by acting as a CDK2 inhibitor [187].

According to some studies, tumor growth can be inhibited by glutamate antagonists. Antiproliferative effects have been observed on lung carcinoma (A549), astrocytoma (MOGGCCM), neuroblastoma (SKNAS), and rhabdomyosarcoma/medulloblastoma (TE671) cells with AMPA receptor antagonists such as GYKI52466, CFM-2, and NBQX [60].

These agents have been studied for their potential to inhibit cancer cell migration and hinder cancer cell growth, with limited side effects on neural function. Ongoing clinical trials are being conducted to further evaluate the efficacy of these agents in targeting neurogenesis in cancer treatment. Moreover, AChR antagonists have also been shown to have a suppressive effect on cancer. Specifically, the M3 mAChR antagonist 4-DAMP has been found to inhibit H82 cell proliferation in a concentration-dependent manner [218]. Recent research suggests that non-small-cell lung carcinoma [219] and small-cell lung carcinoma [220] can be inhibited by ACh antagonists like pirenzepine and darifenacin, respectively.

## 6. Perspectives

The overlooked neuro-microenvironment in cancer has been shown to have a significant impact on cancer progression. Neuronal innervation plays a significant role in both metastasis and primary tumors. Autonomic innervation contributes to the progression of prostate cancer. Compellingly, the two divisions of the autonomic nervous system have distinct functions in prostate cancer.

Compellingly, the two divisions of the autonomic nervous system have distinct functions in prostate cancer. The sympathetic system supports the initial phases of prostate tumor development, while the parasympathetic system facilitates the spread of prostate cancer [18]. Synaptic proximity allows NMDAR signaling to promote brain metastasis [33]. Acetylcholine signaling is involved in lung cancer progression [34]. Tumors can exploit neuronal NMDAR signaling to enhance growth and invasion [62]. Norepinephrine stimulates pancreatic cancer cell proliferation, migration, and invasion through β-adrenergic receptor activation [2]. Nerve fibers infiltrate the tumor microenvironment, which is associated with nerve growth factor production and lymph node invasion in breast cancer [91]. Peripheral nerves participate in the paracrine regulation of pancreatic cancer cell invasion [106]. Glutamate is implicated in the growth and invasion of primary brain tumors and increases pancreatic cancer cell invasion through AMPA receptor activation [125]. Glial cell line-derived neurotrophic factor (GDNF) and integrins contribute to invasion and metastasis in human pancreatic cancer cells [126,169]. L1CAM induces perineural invasion in pancreatic cancer cells [170].

**Figure 5:**
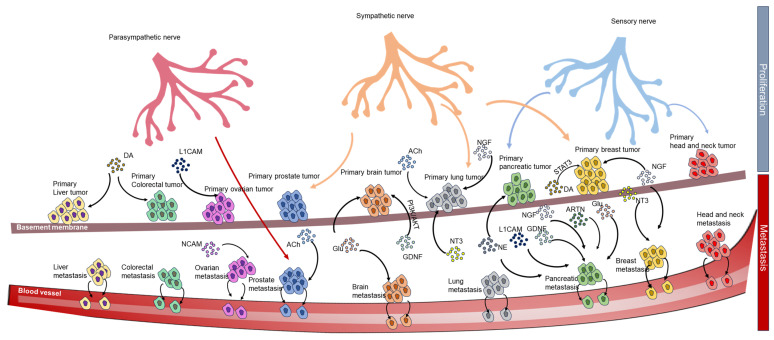
The role of the neuronal innervation of tumors in metastasis versus primary tumors. References for primary liver proliferation: liver tumor [185], colorectal tumor [147], ovarian tumor [108], prostate tumor [18], brain tumor [103,124], lung tumor [19,89,120], pancreatic tumor [2], breast tumor [38,96,125,148], head and neck cancer [45]. References for metastasis: ovarian metastasis [112], prostate metastasis [18,219], breast metastasis [96,125], pancreatic metastasis [90,125,126,169,170,177,178], breast metastasis [91,164]. DA: dopamine; ACh: acetylcholine; NT3: neurotrophin-3; NGF: nerve growth factor; Glu: glutamate; ARTN: artemin; NCAM: neural cell adhesion molecule; L1CAM: L1 cell adhesion molecule. Hh: hedgehog.

Additionally, the neurotrophic factor artemin promotes pancreatic cancer invasion [178]. Muscarinic acetylcholine receptor M1 mediates prostate cancer cell migration and invasion via hedgehog signaling [219]. Furthermore, neurotrophin-3 modulates breast cancer cells and the microenvironment to promote breast cancer brain metastasis [164] (Figure 5).

Moreover, the fact that cancer cells themselves share similar characteristics to neural cells is also noteworthy. Specifically, cancer cells can acquire neuron-like characteristics such as migration, invasion, and proliferation, possibly due to the high expression of many of the same neuroactive substances, neuronal receptors, and proteins, which are involved in normal neurogenesis and cancer progression, explaining why cancer development can be promoted by neuroactive substances released by neurons. Some tumors, such as breast cancer, prostate cancer, lung cancer, and pancreatic cancer, can generate their neuroactive supply through the secretion of some neurotransmitters, such as glutamate and acetylcholine, which promote metastasis and progression (Table 1).

We have suggested that some cancer cell types tend to form similar neural networks to interconnect with one another and transmit signals for cancer cell progression by their own neuroactive substances, aside from the receiving stimulus derived from neurons. Nevertheless, this hypothesis needs more evidence in other cell lines to support it. Based on this hypothesis, we surmised that some drugs, which could inhibit neuronal signals such as anti-psychotic drugs, could be great candidates in cancer treatment as they are able to disrupt neural circuits in cancer by blocking dopamine receptors, AMPA receptors, and acetylcholine receptors. However, this strategy should include measures to minimize the impact on non-cancerous tissues, particularly the CNS. This suggests that we could harness drugs that have a great effect on neuronal targets but find it hard to pass through the blood–brain barrier. It could be useful to mitigate side effects and repurpose some drugs.

**Figure 6:**
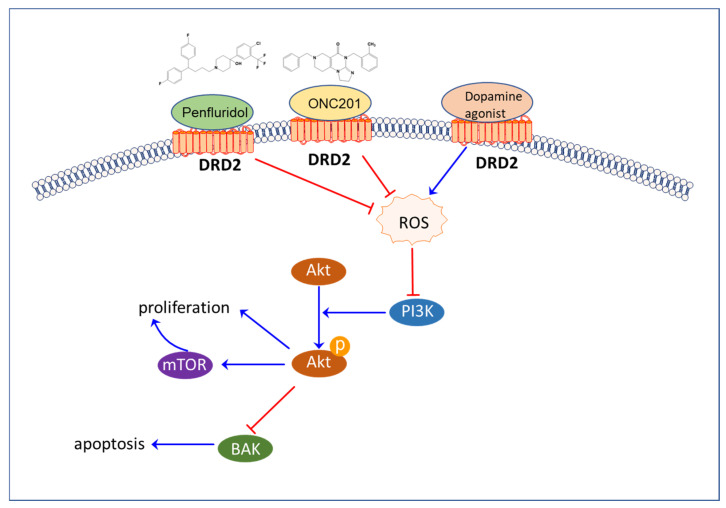
The proposed mechanism by which antipsychotics suppress cancer progression via the DRD2/ROS/PI3K/AKT pathway. Antipsychotics, such as penfluridol and ONC201, target the dopamine receptor D2 (DRD2) and increase ROS levels, which activate the PI3K/AKT pathway. This leads to inhibition of mTOR and the subsequent upregulation of pro-apoptotic proteins such as BAK. Ultimately, this pathway is hypothesized to inhibit cancer self-innervation and prevent cancer progression. DRD2, dopamine receptor D2; ROS, reactive oxygen species; PI3K, phosphoinositide 3-kinases; Akt, Akt serine/threonine kinase; mTOR, mammalian target of rapamycin; BAK, Bcl-2 homologous antagonist/killer.

The similarity between some of the highly expressed proteins and ROS in both neurons and cancer should be taken into account. It could be that the downstream signals of the complex between neuron receptors and their ligands promote the survival, migration and invasion of both neurons and cancer. For example, the ROS/PI3K/Akt/mTOR signaling pathway is involved in both neurogenesis and cancer progression. ROS inducers or antioxidants could be effective inhibitors of neurogenesis in cancer. Antipsychotics targeting the DRD2 receptor, such as penfluridol, pimozide, and ONC201, have been shown to inhibit cancer via ROS induction. It is suggested that ROS/AKT/mTOR could play an important role in neoneurogenesis and could be a potential target for antipsychotic drugs for cancer treatment (Figure 6).

In conclusion, our review suggests that targeting neurogenesis could be a potential avenue for anti-cancer treatment. In particular, we introduced the potential mechanism of neural circuit establishment in cancer as a means for cancer crosstalk and suggested that cancer might respond to neuroactive substances like neurons in order to activate some highly expressed proteins, which can promote neuron and cancer development. Based on that hypothesis, repurposing neuro-targeting drugs and ROS inducers or antioxidants as anti-cancer agents may provide a new strategy for inhibiting neurogenesis in cancer. Future research in this area will expand our understanding of the relationship between neurogenesis and cancer and provide new opportunities for cancer treatment.

## Figures and Tables

**Table 1:** Overview of tumor types, including receptor expression, ligands, innervation, and therapeutic insights. NA: not available.

	Receptors	Ligand	Type of Innervation	Therapeutic Options
Lung cancer	GFRα [97]	ARTN [97]	Sympathetic innervation [19] Cholinergic innervation [121]	NTRK inhibitor [89]
	mAChRs [117]	ACh [120]		
	NMDARs [130]	NA		
	DRD2 [146]	NA		
	Trks [89]	NA		
	NCAM [111]	Small molecules [111]		
Pancreatic cancer	GFRα [97]	ARTN [97,178], GDNF [97,105,168] NRTN [97]	Sensory innervation [23,24]	Beta blocker [2,39] NGF knockdown [23,88] Anti-NT3 [88] DRD2 blocker [221]
	DRD2 [221]	NA		
	Trks [88,90]	BDNF, NT-3, NT-4/5 [88]		
	AMPARs [126], NMDARs [132]	Glutamate [126]		
Prostate cancer	GFRα1 [97,102]	GDNF [97]	Sympathetic innervation [18] parasympathetic innervation [18]	Beta blocker [54,207] β2 and β3 receptor deletion[18] DRD2 blocker [186]
	NMDAR [129,183]	Glutamate [183]		
	Trks [88]	NT-3, BDNF, NGF [88]		
Breast cancer	DRD2 [146]	NA	Sympathetic innervation [38,79] Sensory innervation [91]	Surgical denervation [17] Beta blockers [31,53]
	NMDA [131]	Glutamate [182,183]		
	Trks [89]	BDNF [159], NT-3 [164]		
	GFRα [97,99]	GDNF [97]		
	NGFR [94,96]	NGF [91]		
	L1CAM [107]	NA		
	AChRs [116]	NA		
Gastric cancer	L1CAM [107]	Small molecules [107]	Cholinergic innervation [29] Vagal innervation [27]	Acetylcholine blocker (botulinum toxin A) [27] Surgical denervation [27]
	GFRα [97,104]	NA		
	Trks [29,88]	NGF [29]		
Head and neck cancer	Trks [89]	NA	Autonomic innervation derived from sensory nerves [45]	NA
Skin cancer	Trks [89]	NA	NA	Beta blocker [30]
	L1CAM [107]	Small molecules [107]		
Ovarian cancer	DRD2 [146]	NA	NA	NA
	L1CAM [108]	NA		
	NCAM [112]	NA		
Leukemia	DRD2 [146]	NA	NA	DRD2 blocker [213]
	GFRα [97]	ARTN, NRTN [97]		
Colorectal cancer	GFRα [97]	GDNF [97]	Autonomic innervation [20]	NA
	NA	Ach [119]		
	NMDARs [129]	NA		
	Trks [89]	NA		
Cervical cancer	DRD2 [116]	NA	NA	NA
Esophageal cancer	DRD2 [116]	NA	NA	NA
Liver cancer	L1CAM [107]	Small molecules [107]	NA	DRD1 blocker [185] DRD2 blocker [187]
	DRD1 [185]	Dopamine [185]		
Brain cancer	AMPARs [124,125]	Glutamate [124]	NA	NA
	DRD2 [146]	Dopamine [146]		
	Trks [150,160,165]	NGF [150], NT-3 [165]		
	GFRα [97]	GDNF [97]		
